# Comparison of clinical efficacy of 3D-printed artificial vertebral body and conventional titanium mesh cage in spinal reconstruction after total en bloc spondylectomy for spinal tumors: a systematic review and meta-analysis

**DOI:** 10.3389/fonc.2024.1327319

**Published:** 2024-02-06

**Authors:** Mingjie Dong, Yingjie Gao, Hao Fan, Yushan Wang, Jia Lv, Junjun Bai, Pengfei Shao, Yu Gao, Zhi Lv, Yi Feng

**Affiliations:** ^1^Department of Orthopaedics, the Second Hospital of Shanxi Medical University, Taiyuan, China; ^2^Shanxi Key Laboratory of Bone and Soft Tissue Injury Repair, Taiyuan, China

**Keywords:** 3D-printed artificial vertebral body, titanium mesh cage, spinal tumor, total en bloc spondylectomy, meta-analysis

## Abstract

**Propose:**

This meta-analysis aimed to determine whether 3D-printed artificial vertebral bodies (AVBs) have superior clinical efficacy compared to conventional titanium mesh cages (TMCs) for spinal reconstruction after total en bloc spondylectomy (TES) for spinal tumors.

**Methods:**

Electronic databases, including PubMed, OVID, ScienceDirect, Embase, CINAHL, Web of Science, Cochrane Library, WANFANG, and CNKI, were searched to identify clinical trials investigating 3D-printed AVB versus conventional TMC from inception to August 2023. Data on the operation time, intraoperative blood loss, preoperative and postoperative visual analogue scale (VAS) scores, preoperative and postoperative Frankel classification of spinal cord injury, vertebral body subsidence, and early complications were collected from eligible studies for a meta-analysis. Data were analyzed using Review Manager 5.4 and Stata 14.0.

**Results:**

Nine studies assessing 374 patients were included. The results revealed significant differences between the 3D-printed AVB and conventional TMC groups with regard to operation time (*P* = 0.04), intraoperative blood loss (*P* = 0.004), postoperative VAS score (*P* = 0.02), vertebral body subsidence (*P* < 0.0001), and early complications (*P* = 0.02). Conversely, the remaining preoperative VAS score and Frankel classifications (pre-and postoperative) did not differ significantly between the groups.

**Conclusion:**

The 3D-printed AVB in spinal reconstruction after TES for spinal tumors has the advantages of a short operative time, little intraoperative blood loss, weak postoperative pain, low occurrence of vertebral body subsidence and early complications, and a significant curative effect. This could provide a strong basis for physicians to make clinical decisions.

**Systematic review registration:**

https://www.crd.york.ac.uk/prospero/display_record.php?ID=CRD42023441521, identifier CRD42023441521.

## 1 Introduction

In recent years, the incidence of spinal tumors has been rising (1). Regardless of whether primary or metastatic spinal tumors are present, they can progressively develop and compress the spinal cord or cauda equina nerve, leading to neurological dysfunction, which can cause spinal instability, resulting in intractable pain and seriously affecting the quality of life. As the most important surgical method for spinal tumors, the main purpose of total en bloc spondylectomy (TES) is to completely remove the diseased tissues and reconstruct the integrity and stability of the spine (2, 3). In conventional methods, titanium mesh cages (TMCs) composite bone graft materials are commonly used for spinal reconstruction; however, they are prone to poor fusion of vertebral bodies and have a high probability of subsidence, which leads to the failure of internal fixation (4). Both Shinmura et al. (5) and Ji et al. (6) found that due to the cutting effect of the TMC, subsidence was prone to occur, and the incidence rate is as high as 42.5–79.7%, which led to the reconstruction failure. Autologous bone reconstruction has problems such as limited sources and secondary injuries, while allogeneic bone has risks of bone nonunion, infection, rejection, etc. (7). The 3D-printed technology appeared in the mid-1990s and has been widely used in many medical fields (8, 9). In the field of spinal surgery, 3D-printed artificial vertebral bodies (AVBs) have a personalized design that can achieve a complete match and closely fit the upper and lower vertebral bodies, reducing the probability of subsidence (10). Because 3D-printed AVB can simulate the normal human anatomical structure, its reconstruction after spinal tumor resection has more biomechanical characteristics than conventional TMC, and the prosthesis is made of titanium alloy, which has good biocompatibility (11).

Currently, owing to the short clinical application time of 3D-printed AVB, its efficacy and safety lack evidence-based medical evidence. The meta-analysis aimed to compare the clinical efficacy and safety of 3D-printed AVB and conventional TMC in spinal reconstruction after total en bloc spondylectomy for spinal tumors, and to evaluate their advantages. We hypothesized that the clinical efficacy and safety of the 3D-printed AVB were superior to those of the conventional TMC group.

## 2 Methods

This meta-analysis was conducted in accordance with the Preferred Reporting Items for Systematic Reviews and Meta-Analyses (PRISMA) statement guidelines.

### 2.1 Search strategy

In accordance with the recommendations of the Cochrane Collaboration, several comprehensive databases were retrieved for studies, including PubMed, OVID, ScienceDirect, Embase, CINAHL, Web of Science, Cochrane Library, WANFANG, and CNKI, were searched from inception to June 2023. Grey literature was identified via manual searches of journal catalogs and references. All relevant studies were retrieved without language restrictions and, if necessary, translated. The search was conducted by using the keywords, “Printing, Three-Dimensional,” “Artificial Vertebral Body,” “Titanium Mesh Cage,” “Spine,” “Neoplasms,” and “Total En Bloc Spondylectomy.” The search strategy was performed by using the all fields, i.e., “((3D-printed artificial vertebral body) OR (Three-Dimensional-printed artificial vertebral body) OR (Titanium Mesh Cage)) AND ((spinal neoplasms) OR (spinal tumors)) AND (total en bloc spondylectomy).” Relevant studies and abstracts were manually searched to identify those that met the inclusion search.

### 2.2 Study selection

Based on the initially retrieved abstracts, two reviewers independently selected the relevant studies that met the established criteria for a comprehensive review. The full text of the study was reviewed if the abstract did not provide the full information. Studies meeting the established criteria were identified and included.

The inclusion criteria were (1) patients diagnosed with spinal tumors based on pathological results for any age, sex, or race (2); patients undergoing total en bloc spondylectomy (3); implants with 3D-printed AVBs or TMCs (4); controlled trials, cohort studies, prospective studies, and retrospective studies; and (5) results reporting one or more of the operative time, intraoperative blood loss, visual analogue scale (VAS) scores, Frankel classification, vertebral body subsidence, and early complications. The exclusion criteria were as follows (1): cadaveric studies (2); duplicate publications (3); reviews, case reports, letters, commentaries, editorials, or expert opinions (4); studies with missing or incomplete outcome data; and (5) studies not available in the full text.

### 2.3 Data extraction and quality assessment

Based on a pre-established data extraction form, two investigators (M. D and YJ. G) independently extracted the data from the included studies and resolved any disagreements by consulting a third reviewer (Y. F).

The following information was extracted (1) the basic characteristics of the included studies, such as the authors, country, publication date, article title, and journal title (2); the methodological characteristics of the studies: randomized, controlled, and blinded (3); demographic characteristics such as race, sex, and age; and (4) other parameters, such as sample size, tumor location, tumor type, pathological diagnosis, segment, surgical approach, follow-up time, and outcomes. We attempted to communicate with the corresponding authors while relevant information about the included studies was unclear or missing and could not be analyzed. The general characteristics of these included nine studies are presented in **Table 1**. The methodological quality of the included studies was assessed using the risk-of-bias assessment tool outlined in the Cochrane Handbook. Seven domains were evaluated (1): random sequence generation (2); allocation concealment (3); blinding of participants and personnel (4); blinding of outcome assessment (5); incomplete outcome data (6); selective reporting; and (7) other biases.

**Table 1:** Details of the included studies.

First author	Year	Mean age (years)		Sample size (M/F)		Tumor location (C/T/L)		Tumor type (P/S)		Segment (1/2/3/Multiple)		Approach (C/T, L)	Follow-up (months)	Outcomes
		AVB	TMC	AVB	TMC	AVB	TMC	AVB	TMC	AVB	TMC			
Chen et al. (12)	2023	47.62	46.13	12/18	14/16	2/21/7	1/24/5	30/0	30/0	0/12/18/0	0/16/14/0	A/Po	3	OT, BL, VAS, OC
Hu et al. (13)	2022	38.2	43.3	7/11	10/3	18/0/0	13/0/0	18/0	13/0	–	–	A+Po/-	12	OT, BL, OC
Ji et al. (14)	2020	54.50	64	15/6	10/2	0/17/4	0/12/0	5/16	1/11	18/2/1/0	12/0/0/0	-/Po	10.9	OT, BL, VAS, VBS
Li et al. (15)	2019	42	37.08	4/4	4/6	0/5/3	0/7/3	7/1	7/3	2/1/4/1	4/3/3/0	-/Po or A+Po	20.5	OT, BL, Frankel, OC
Qing et al. (16)	2020	38.64	38.93	19/13	20/25	12/11/9	12/15/ 18	10/22	14/31	–	–	Po/Po	6	OT, BL, VAS, Frankel, OC
Wang L et al. (17)	2021	55.20	52.40	14/10	13/11	2/15/7	1/19/4	0/24	0/24	24/0/0/0	24/0/0/0	A/Po	20.4	OT, BL, VAS, OC
Wang X et al. (18)	2021	40.50	40.68	13/11	18/13	0/-/-	0/-/-	–	–	–	–	-/Po	12	OT, BL, VAS, Frankel, VBS, OC
Zhang et al. (19)	2021	43.30	45.40	10/4	8/6	0/8/6	0/10/4	13/1	10/4	12/0/2/0	13/1/0/0	-/Po	16.8	OT, BL, VAS, Frankel, VBS, OC
Zhou et al. (20)	2023	45.56	50.27	4/5	9/6	9/0/0	15/0/0	9/0	15/0	–	–	A+Po/-	24	VBS

AVB, 3D-printed artificial vertebral body; TMC, titanium mesh cage; M, male; F, female; C, cervical; T, thoracic; L, lumbar; P, primary; S, secondary; A, anterior approach; Po, posterior approach; OT, operation time (min); BL, intraoperative blood loss (ml); VAS, visual analogue scale score; VBS, vertebral body subsidence; OC, operative early complications.

### 2.4 Outcome measures

The outcome measures mainly included operation time, intraoperative blood loss, preoperative and postoperative VAS scores, preoperative and postoperative Frankel classifications (grades A–E were scored 1–5 points, respectively), vertebral body subsidence (height > 3 mm), and early complications.

### 2.5 Statistical analysis

Continuous outcomes in the included studies were expressed as weighted mean differences (WMDs) or standard mean differences (SMDs), and odds ratios (ORs) were used for dichotomous outcomes, all with 95% confidence intervals (CIs). The WMD was mainly used to express the synthetic data of the VAS scores and Frankel classification. The SMD was used to express the operation time and intraoperative blood loss because of the use of different testing methods or too large of these means. Moreover, the OR was used to express vertebral body subsidence and early complications. Heterogeneity was determined by evaluating the proportion of inconsistencies among studies that were caused by actual differences among studies, not random errors or by chance (21). Heterogeneity was detected mainly by *I²* and chi-square tests in this study. When significant heterogeneity was found (*P* ≤ 0.10, *I²* > 50%), a random-effects model was chosen, and when there was no statistical evidence of heterogeneity (*P* > 0.10, *I²* ≤ 50%), a fixed-effects model was used (22). Sensitivity and subgroup analyses were performed by excluding individual studies or factors when heterogeneity was found. Moreover, we checked the related factors by meta-regression analysis to search for the source, and by funnel plots and Egger’s test to estimate the publication bias. When heterogeneity was not identified, the results were qualitatively analyzed. The difference was considered statistically significant at *P* ≤ 0.05. All data were analyzed using Review Manager 5.4 and Stata 14.0 statistical software provided by the Nordic Cochrane Centre and the Stata Corp, respectively.

## 3 Results

### 3.1 Search and selection

**Figure 1:**
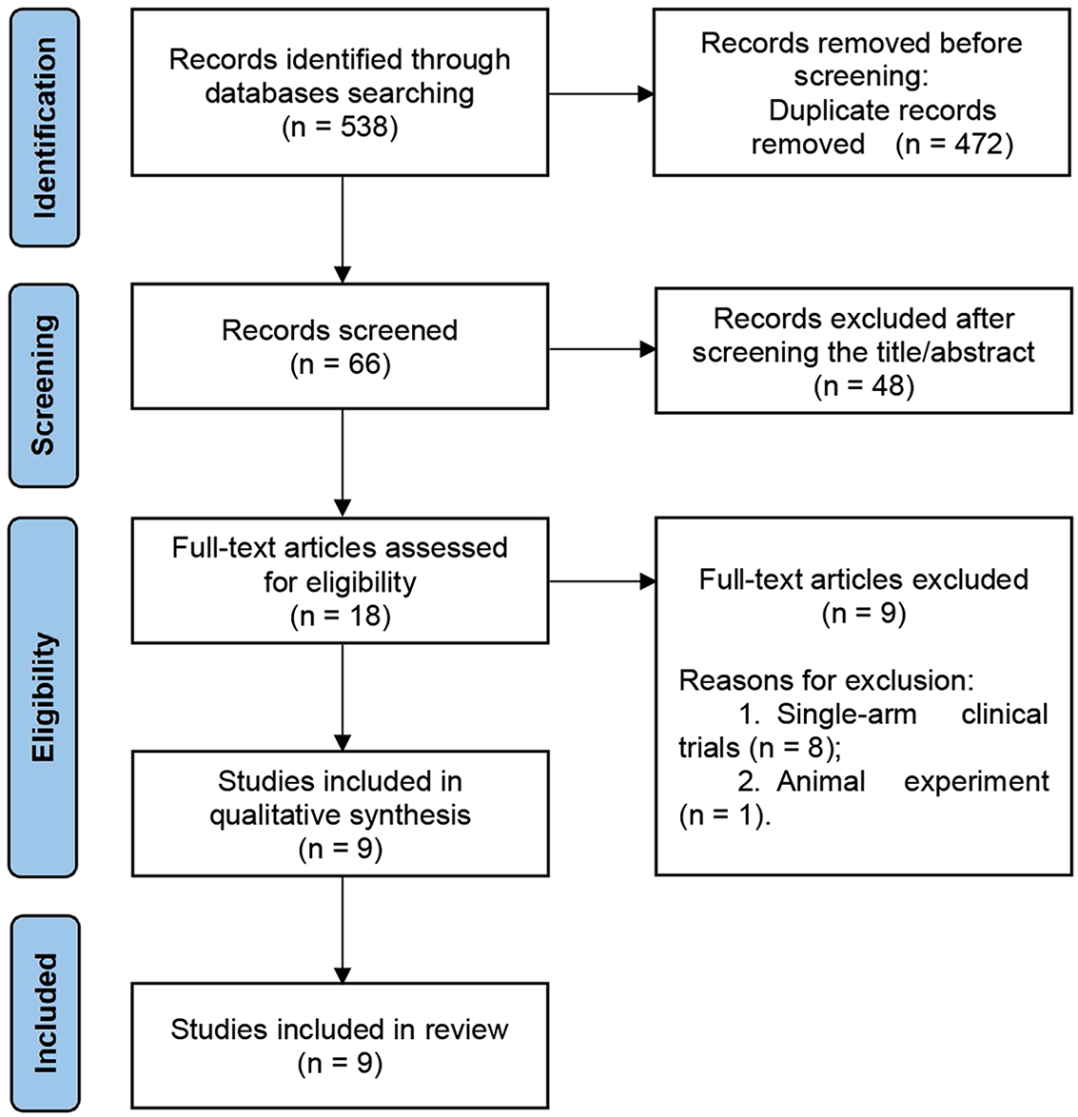
Flow diagram of the study search strategy.

**Figure 2:**
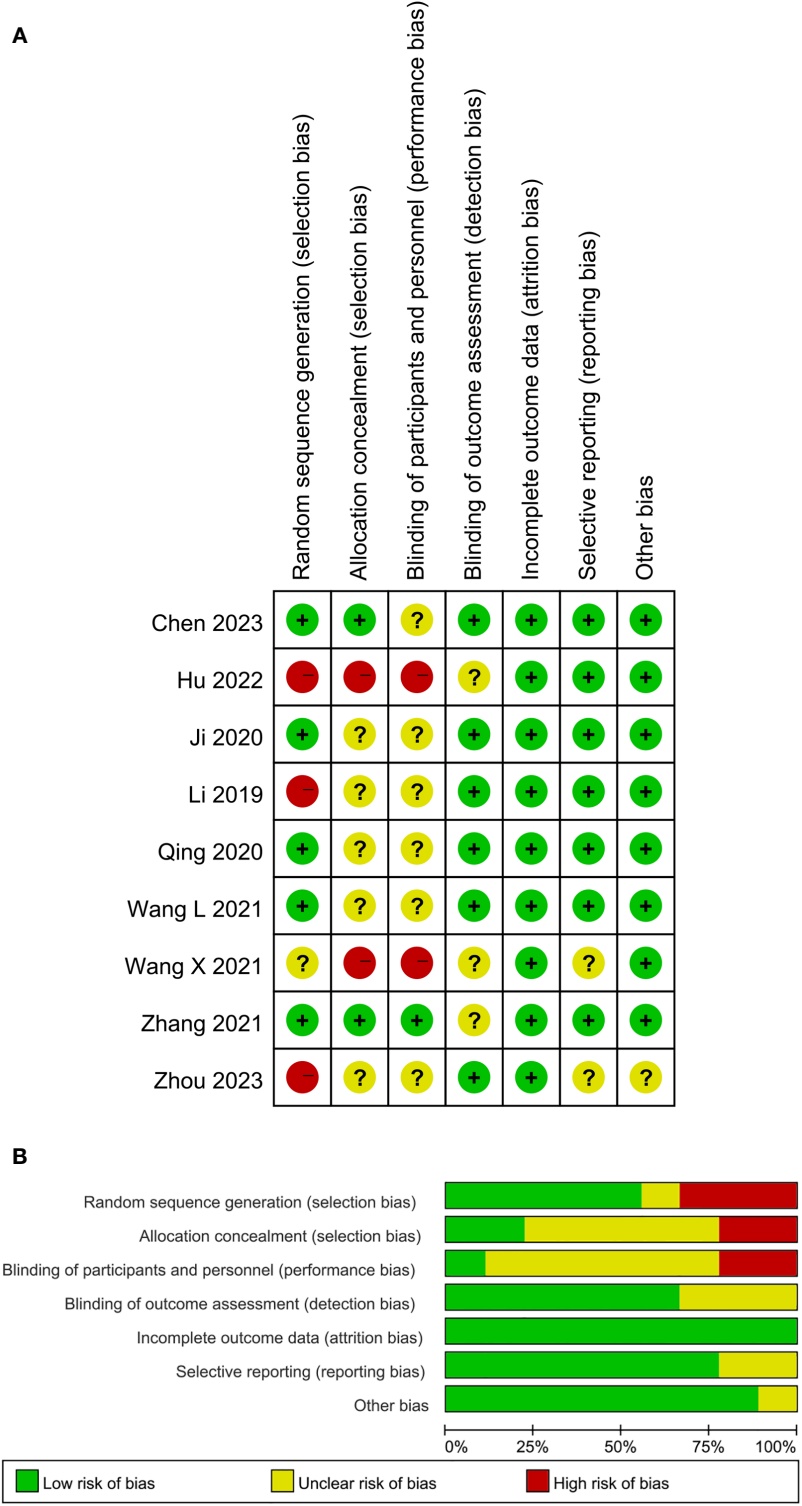
**(A)** Risk of bias summary of the included studies. **(B)** Risk of bias graph.

Through online and manual searches, 538 articles were initially identified. Sixty-six studies remained after removing 472 duplicate studies. Of these, 48 were excluded after reading the abstracts because they were irrelevant to the purpose of this study. The remaining 18 studies were read in full, of which nine were excluded as single-arm clinical trials or animal experiments. Ultimately, nine studies (one in English and eight in Chinese) involving 374 patients were included (12–20). A Flow Diagram of the studies selection process based on the PRISMA 2020 flow diagram is shown in **Figure 1** (23). The quality of methodology in the included studies was high, with a low likelihood of bias. A summary and graphs of the risk of bias are shown in **Figure 2**.

### 3.2 Operation time

**Figure 3:**
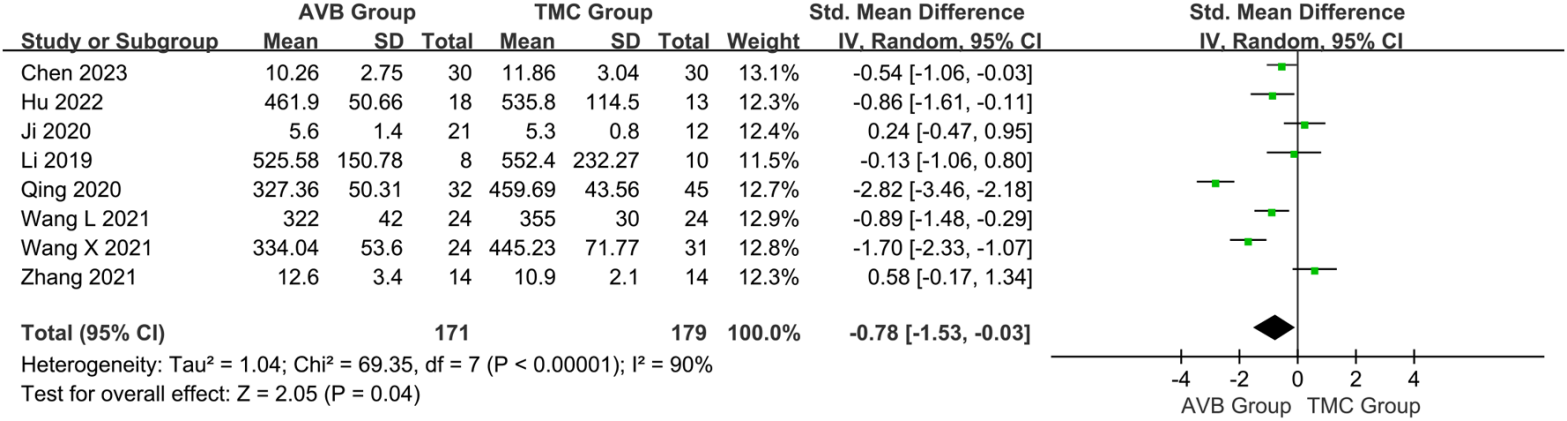
Forest plot of the operation time in the two groups.

Eight of the included studies (12–19) compared the operation time between 3D-printed AVB (n = 171) and conventional TMC (n = 179) groups. A random-effects model was employed for this parameter because the heterogeneity among the studies was significant (*P* < 0.00001, *I²* = 90%). The standard mean difference in the operation time between two groups was -0.78, significantly inclined toward the conventional TMC group (95% CI: -1.53 to -0.03, *P* = 0.04, **Figure 3**). No sources of heterogeneity were identified after the sensitivity and meta-regression analyses (**Tables 2**, **3**). Therefore, a qualitative descriptive analysis was performed, and the results showed that four studies (12, 16–18) reported that the operation time of the 3D-printed AVB group was significantly shorter than that of the conventional TMC group, whereas the remaining four studies (13–15, 19) reported no difference between the two groups.

**Table 2:** Sensitivity analysis.

Study	Parameters	Before exclusion	After exclusion	Statistical significance
Any study	OT	SMD = -0.78, 95% CI = -1.53 to -0.03, *P* < 0.00001, *I²* = 90%	*P* ≤ 0.1, *I²* > 50%	No difference
Any study	BL	SMD = -2.38, 95% CI = -3.82 to -0.93, *P* < 0.00001, *I²* = 96%	*P* ≤ 0.1, *I²* > 50%	No difference

SMD, standard mean difference; CI, confidence interval; OT, operation time; BL, intraoperative blood loss.

**Table 3:** Meta-regression analysis in operation time and intraoperative blood loss ratio between two groups.

Parameters	Variable	Coefficient	Standard error	*P* value	95% confidence interval
OT	Age	-0.057	0.067	0.549	-0.905 to 0.791
	Sex	2.096	0.727	0.213	-7.146 to 11.339
	Year	0.247	0.247	0.500	-2.890 to 3.384
	Follow up	0.373	0.080	0.134	-0.638 to 1.384
	Type	-3.461	0.742	0.134	-12.885 to 5.963
	Location	2.244	0.734	0.201	-7.082 to 11.570
	Constant	-503.531	498.550	0.497	-6838.209 to 5831.147
BL	Age	0.214	0.937	0.857	-11.692 to 12.121
	Sex	2.839	10.108	0.826	-125.591 to 131.268
	Year	0.805	3.269	0.846	-40.728 to 42.338
	Follow up	0.984	1.119	0.541	-13.231 to 15.199
	Type	-8.020	10.865	0.595	-146.067 to 130.027
	Location	4.127	10.480	0.761	-129.033 to 137.286
	Constant	-1650.589	6600.934	0.844	-85523.400 to 82222.220

OT, operation time; BL, intraoperative blood loss.

### 3.3 Intraoperative blood loss

**Figure 4:**
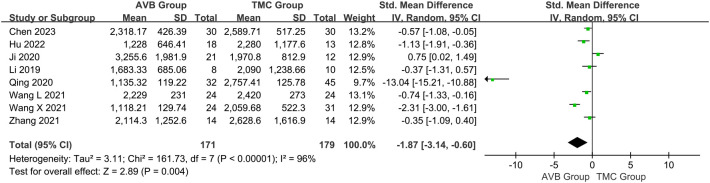
Forest plot of the intraoperative blood loss in the two groups.

Eight studies compared the intraoperative blood loss between the 3D-printed AVB (n = 171) and conventional TMC (n = 179) groups (12–19). A random-effects model was employed for this parameter because the heterogeneity among the studies was significant (*P* < 0.00001, *I²* = 96%). The standard mean difference in blood loss between two groups was -1.87, significantly inclined toward the conventional TMC group (95% CI: -3.14 to -0.60, *P* = 0.004, **Figure 4**). No sources of heterogeneity were identified after the sensitivity and meta-regression analyses (**Tables 2**, **3**). Therefore, a qualitative descriptive analysis was performed, and the results showed that five studies (12, 13, 16–18) reported significantly less intraoperative blood loss in the 3D-printed AVB group than in the conventional TMC group, whereas the remaining three studies (14, 15, 19) reported no significant difference between the two groups.

### 3.4 VAS scores

**Figure 5:**
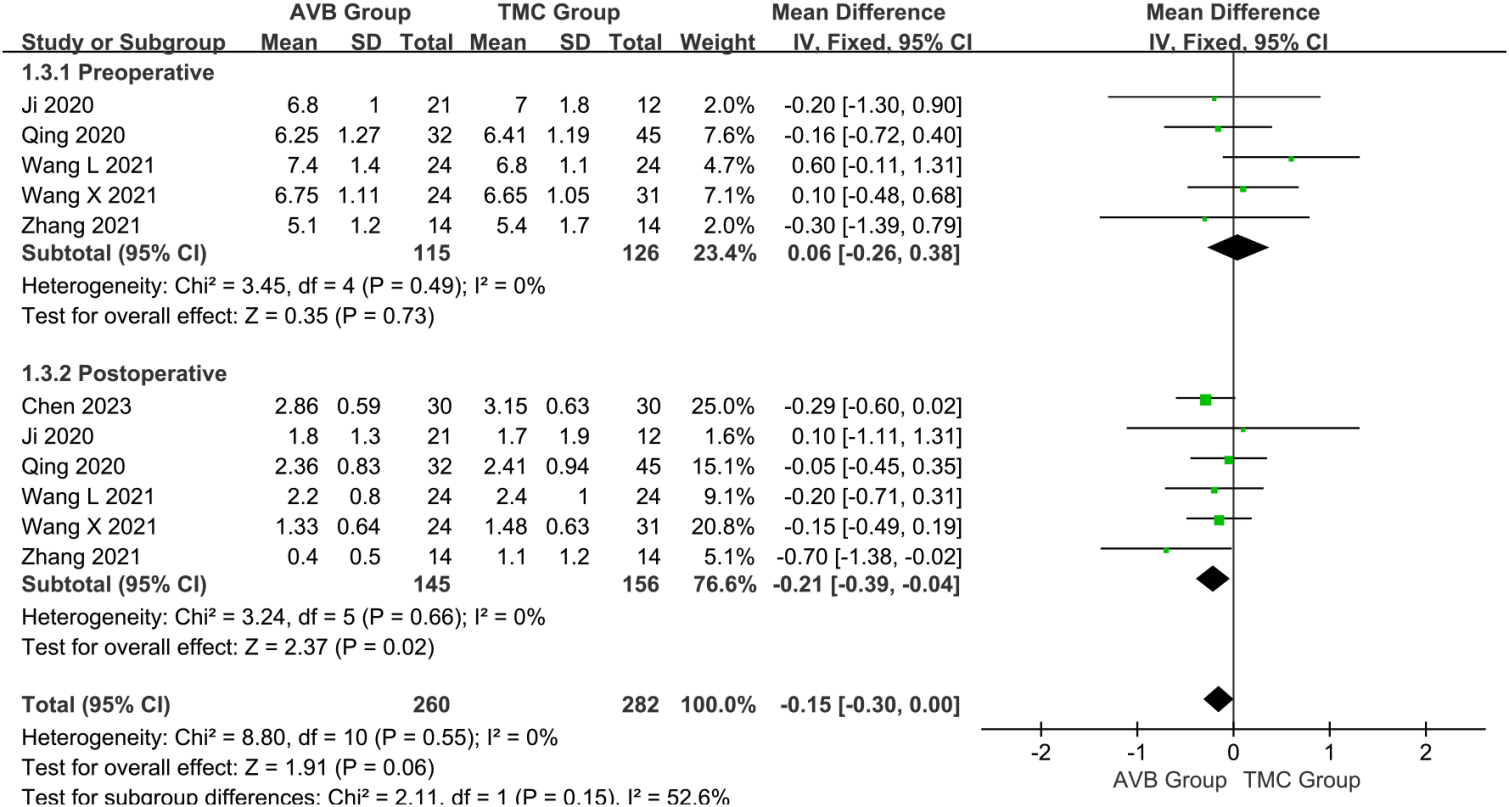
Forest plot of the preoperative and postoperative visual analogue scale scores in the two groups.

The VAS scores were subclassified according to follow-up time: preoperative and postoperative scores. The preoperative VAS score between 3D-printed AVB (n = 115) and conventional TMC (n = 126) groups was compared in five studies (14, 16–19). The results showed that the 3D-printed AVB group had a higher preoperative VAS score than that in the conventional TMC group, but the difference was also not statistically significant (WMD = 0.06, 95% CI: -0.26 to 0.38, *P* = 0.73; *I²* = 0%, fixed-effects model; **Figure 5**). Six studies (12, 14, 16–19) compared the postoperative VAS score between the 3D-printed AVB (n = 145) and conventional TMC (n = 156) groups. However, the results showed that the 3D-printed AVB group achieved a significantly lower postoperative VAS score than that achieved by the conventional TMC group (WMD = -0.21, 95% CI: -0.39 to -0.04, *P* = 0.02; *I²* = 0%, fixed-effects model; **Figure 5**).

### 3.5 Frankel classification

**Figure 6:**
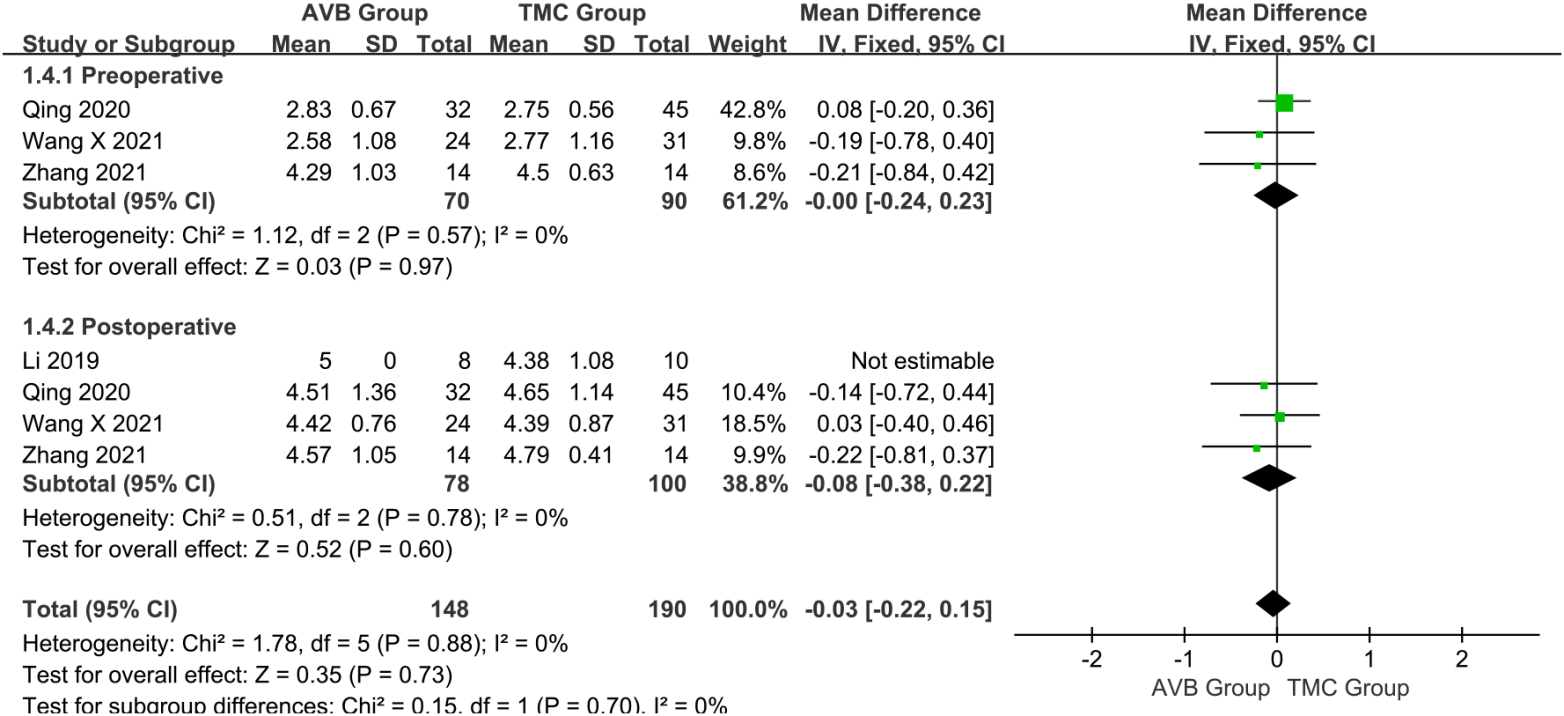
Forest plot of the preoperative and postoperative Frankel classification in the two groups.

The Frankel classification was divided into: before and after surgery. Three studies (16, 18, 19) compared the preoperative Frankel classification between the 3D-printed AVB (n = 70) and conventional TMC (n = 90) groups. The results showed that the preoperative Frankel classification in the 3D-printed AVB group was the same as that in the conventional TMC group (WMD = 0, 95% CI: -0.24 to 0.23, *P* = 0.97; *I²* = 0%, fixed-effects model; **Figure 6**). The postoperative Frankel classification between the 3D-printed AVB (n = 78) and conventional TMC (n = 100) groups was compared in four studies (15, 16, 18, 19). The weighted mean difference in the postoperative Frankel classification between the two groups was -0.08, which non-significantly inclined toward the conventional TMC group (95% CI: -0.38 to 0.22, *P* = 0.60; *I²* = 0%, fixed-effects model; **Figure 6**).

### 3.6 Vertebral body subsidence

**Figure 7:**
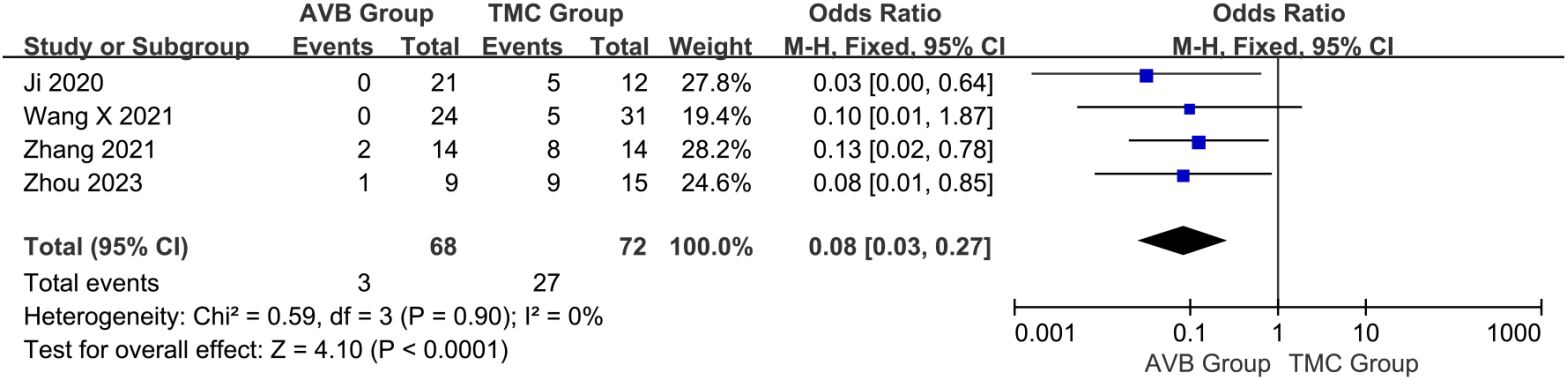
Forest plot of the occurrence of vertebral body subsidence in the two groups.

The occurrence of vertebral body subsidence between 3D-printed AVB (n = 68) and conventional TMC (n = 72) groups was compared in four studies (14, 18–20). The OR in vertebral body subsidence between two groups was 0.08, significantly inclined toward conventional TMC group (95% CI: 0.03 to 0.27, *P* < 0.0001; *I²* = 0%, fixed-effects model; **Figure 7**).

### 3.7 Early complications

**Figure 8:**
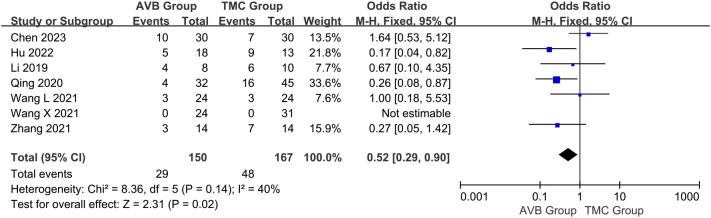
Forest plot of the occurrence of early complications in the two groups.

Early complications were mainly those that occurred during the surgery or during the recovery period after the surgery. Early complications mainly included cerebrospinal fluid leakage, pleural effusion or lung infection due to pleural rupture, poor healing of infected wounds, and neurological complications. The occurrence of early complications between 3D-printed AVB (n = 150) and conventional TMC (n = 167) groups was compared in seven studies (12, 13, 15–19). The OR in early complications between two groups was 0.52, significantly inclined toward the conventional TMC group (95% CI: 0.29 to 0.90, *P* = 0.02; *I²* = 40%, fixed-effects model; **Figure 8**).

### 3.8 Sensitivity and subgroup analyses

The results showed that the heterogeneity of the operation time and intraoperative blood loss was high. A random-effects model was employed to partially eliminate the effect of heterogeneity, but it was still high. After removing each included article separately for operation time and intraoperative blood loss (**Table 2**), we found that heterogeneity did not decrease significantly, but it did not affect the results, which shows that the results of this meta-analysis are relatively reliable. And we conducted a qualitative descriptive analysis of operation time and intraoperative blood loss.

In addition, the tumor pathologies and types in the nine included studies are presented in **Supplementary Table 1**. Based on the pathological tumor diagnosis, we divided these studies into Type 1 (primary), Type 2 (primary and metastatic), and Type 3 (metastatic) for subgroup analysis to eliminate heterogeneity.

**Figure 9:**
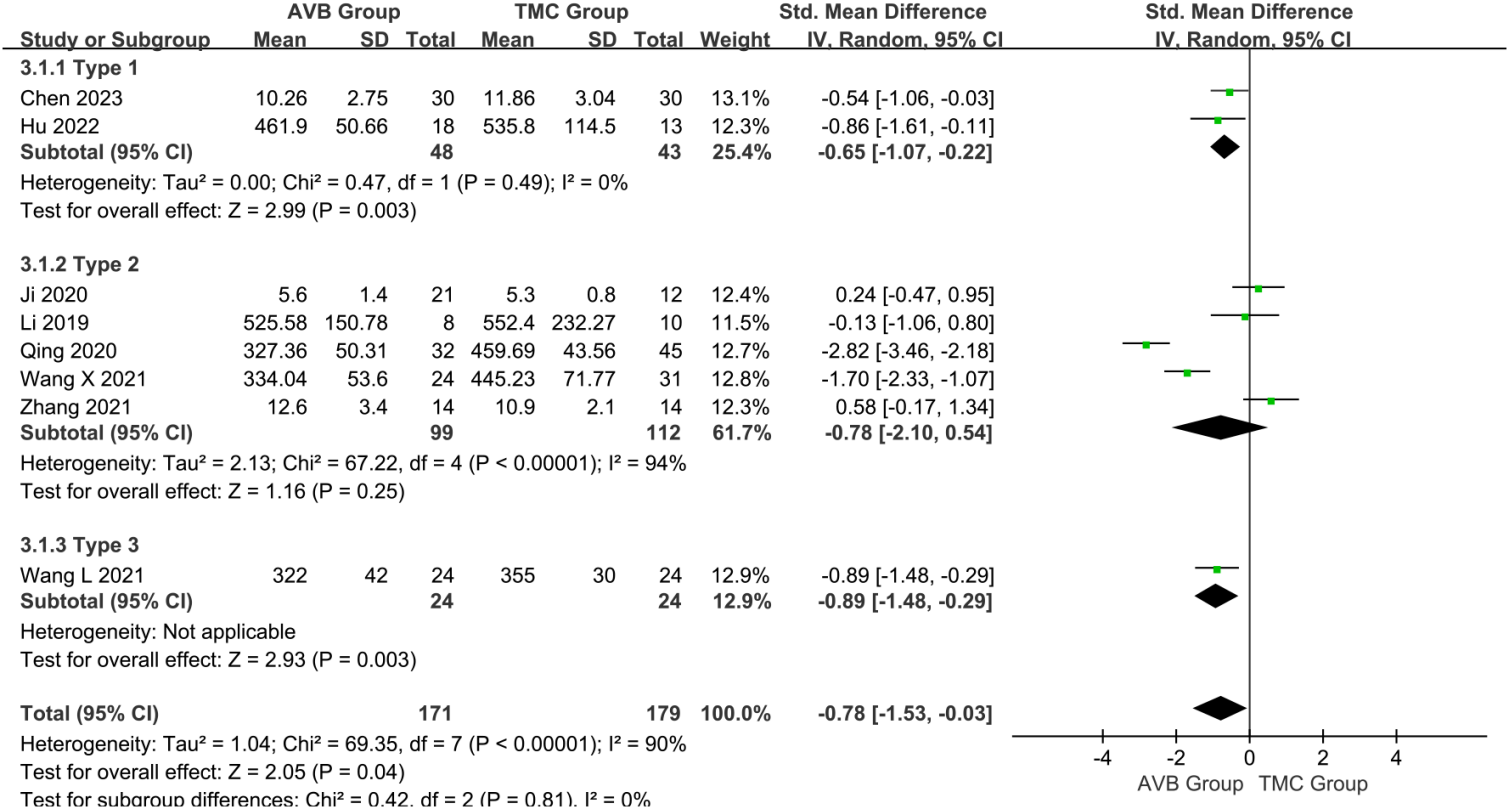
Forest plot of subgroup analysis of the operative time in the two groups.

**Figure 10:**
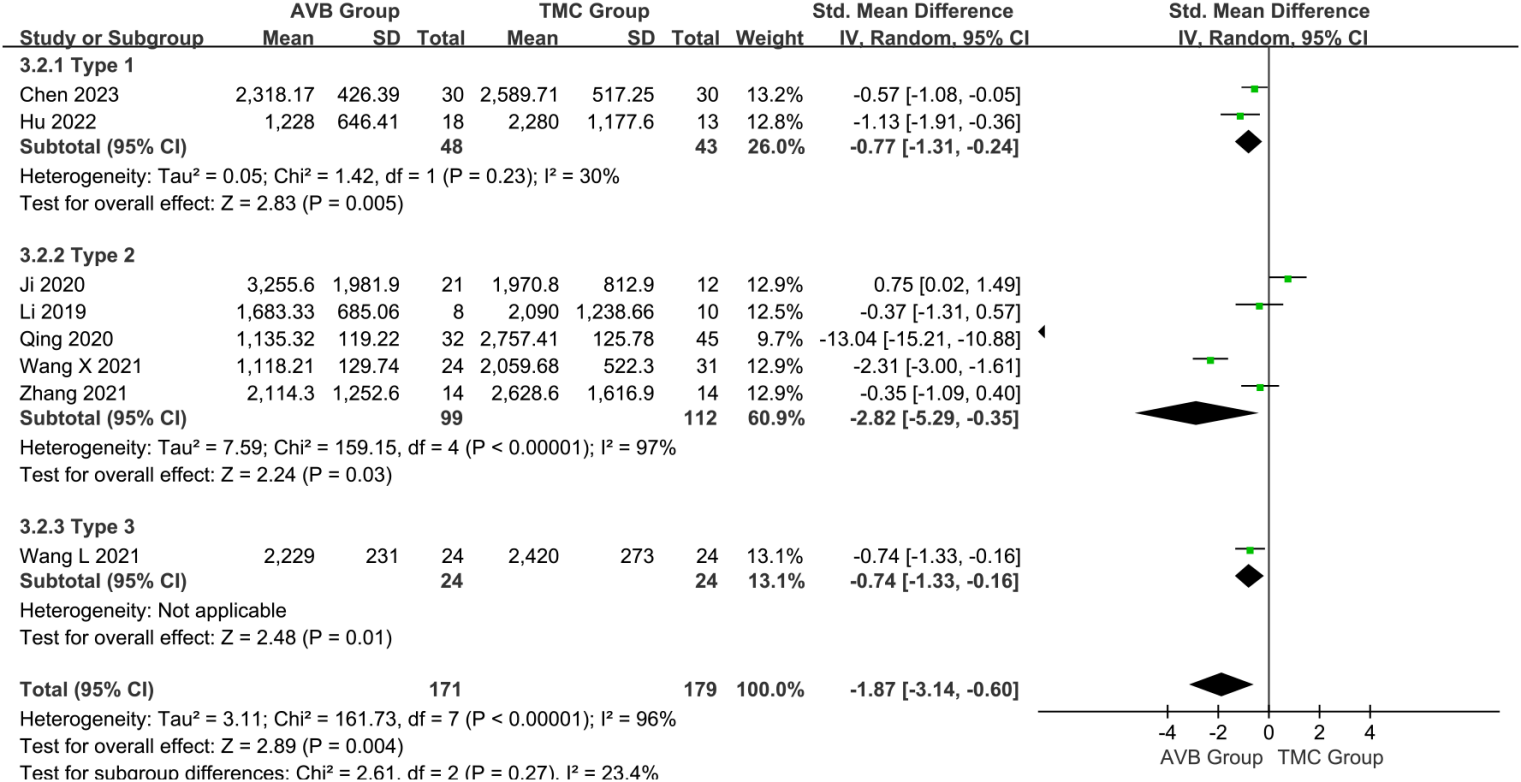
Forest plot of subgroup analysis of the intraoperative blood loss in the two groups.

For the operation time Type 2 subgroup, a random-effects model was employed because the heterogeneity among the studies was still significant (*P* < 0.00001, *I²* = 94%). The standard mean difference in this subgroup between the two groups was -0.78, which non-significantly inclined toward the conventional TMC group (95% CI: -2.10 to 0.54, *P* = 0.25; **Figure 9**). For the intraoperative blood loss Type 2 subgroup, a random-effects model was employed because the heterogeneity among the studies was still significant (*P* < 0.00001, *I²* = 97%). The standard mean difference in this subgroup between the two groups was -2.82, significantly inclined toward the conventional TMC group (95% CI: -5.29 to -0.35, *P* = 0.03; **Figure 10**). The subgroup analysis did not eliminate heterogeneity. Qualitative descriptive analysis showed that two studies (16, 18) reported significantly shorter operation time and less intraoperative blood loss in the 3D-printed AVB group than in the conventional TMC group, whereas the remaining three studies (14, 15, 19) reported no difference between the two groups.

### 3.9 Meta-regression analysis

**Table 3** shows the results of the meta-regression analysis of the operation time and intraoperative blood loss between the 3D-printed AVB and conventional TMC groups in terms of age, sex, publication year, follow-up time, tumor type, and location. The results showed that these variables did not significantly affect the mean difference in operation time or intraoperative blood loss between the two groups (all *P* > 0.05).

### 3.10 Publication bias analysis

The results of the publication bias analysis are shown in **Table 4** and **Supplementary Figures 1–8**. We constructed funnel plots to evaluate publication bias (**Supplementary Figures 1–8**), and the results were largely symmetrical except for intraoperative blood loss, indicating that publication bias was acceptable in our analysis. The Egger’s test was performed for further evaluation (**Table 4**). The results revealed that publication bias existed in terms of the intraoperative blood loss between the two groups (*P* = 0.032).

**Table 4:** Publication bias analysis (Egger’s test) in operation time and intraoperative blood loss between two groups.

Parameters	Standard effect	Coefficient	Standard error	*P* value	95% confidence interval
OT	slope	-2.670	2.410	0.310	-8.567 to 3.227
	bias	5.346	7.125	0.481	-12.088 to 22.780
BL	slope	2.859	1.471	0.100	-0.741 to 6.458
	bias	-10.701	3.840	0.032	-20.096 to -1.306

OT, operation time; BL, intraoperative blood loss.

## 4 Discussion

With the in-depth studies of spinal tumors and the development of adjuvant therapy, the survival time of patients has been significantly prolonged, greatly increasing the chances of surgical intervention. Two main points must be considered in the surgical treatment of spinal tumors: total en bloc resection of the tumors, which can reduce local recurrence, and spinal reconstruction. Tomita et al. (24) proposed TES as an important surgical method for the en bloc resection of spinal tumors. The Weinstein-Boriani-Biagini (WBB) classification can describe the anatomical location of spinal tumors, growth depth of tumors around the spinal cord, and invasion of soft tissue around the spine, as well as personalized surgical resection guidance for spinal tumors of different shapes (25). Compared with the TES, en bloc resection under the guidance of the WBB classification increases the difficulty of the surgical operation; however, it can formulate a more stringent tumor-free osteotomy plan (26). Because few studies on this procedure met the inclusion criteria, our results focused mainly on patients undergoing TES.

The most widely used reconstruction method is TMC combined with autologous or allograft bone (27). As emerging implants for spinal reconstruction, 3D-printed AVB offer advantages in conformal matching and osseointegration (28). After including nine studies, our results confirmed that 3D-printed AVB was superior to conventional TMC in terms of reducing operation time and intraoperative blood loss, reducing postoperative VAS score, and reducing the occurrence of vertebral body subsidence and early complications, which supports the hypothesis that the clinical efficacy and safety in the 3D-printed AVB group were superior to those in the conventional TMC group.

The Ti6Al4V is an important material for 3D-printed AVB because of its excellent mechanical properties and tissue biocompatibility (29). By the microporous structure loading nano-zinc oxide composite hydroxyapatite, antibiotics, and anti-tumor drugs, it can elevate osseointegration between the host bone and implants and prevent infection and tumor recurrence (30, 31). The 3D-printed AVBs in the nine included studies were fabricated from Ti6Al4V. Recently, Wang et al. (32) and Guo et al. (33) found that compared with Ti6Al4V implants, porous tantalum implants with a pore size of 400 μm had better cell adhesion and proliferation; radiological analyses showed that the new bone formation rate of tantalum implants was significantly higher; mechanical analyses showed that tantalum implants had controllable elastic modulus and compressive strength. Tantalum is expected to become the focus for 3D-printed AVB metals in the future. Although 3D-printed AVB have been widely used clinically, most available studies involved early efficacy evaluations and long-term follow-up studies of large-scale cases are lacking.

The spinal tumor type, location, size, number of vertebral bodies involved, surgical approach, and operator experience all impact the operation time and intraoperative blood loss (34). The richness of the tumor blood supply and whether preoperative vascular embolization therapy can also cause changes in intraoperative blood loss. Zhou et al. (35) believed that in traditional surgery, operators repeatedly endeavored to select suitable interbody fusion cages, which increased the operation time and intraoperative blood loss. In 3D-printed AVB surgery, the entire vertebral body is scanned and modeled by CT or MRI before surgery, thus avoiding the repeated selection of suitable interbody fusion cages, shortening the operation time, and reducing the blood loss (36). Our results showed that both operation time and blood loss were significantly different between the two groups; however, the reliability of the results was questionable because of the high heterogeneity among the studies. Therefore, whether implanting a 3D-printed AVB for spinal reconstruction during TES can significantly shorten the operation time and reduce intraoperative blood loss requires further verification.

The subsidence of implants can easily lead to the loss of spinal height and physiological curvature, adjacent vertebral fracture, or nail-rods fracture, etc., resulting in severe instability of the spine. Therefore, reducing the occurrence of implants subsidence is key to maintaining the long-term stability of spinal reconstruction. When the subsidence height is greater than 5 mm, the fixation system is prone to fracture due to overstress, resulting in the failure of vertebral body reconstruction (27). The main reason for this phenomenon is that the elastic moduli of conventional TMC are not consistent with those of normal human bones, leading to a stress shielding effect. Zhang et al. (19) noticed that the stress shielding effect could cause the loss of adjacent vertebral bone tissue and osteoporosis, resulting in the displacement of implants, formation of an angle with the endplate of the adjacent vertebral body, and even fracture of implants. Excessive stress and sharp edges can also cause adjacent vertebral fractures, directly destroying spinal stability (27). Our study found that the incidence of vertebral body subsidence was lower in the 3D-printed AVB group than that in the conventional TMC group. The 3D-printed AVB was designed according to the location of the tumor and physiological curvature of the spine. The large contact area is conducive to the adhesion of bone tissue cells and provides good stability (37). Its three-dimensional microporous structure renders it high in strength and low in modulus, and it is less prone to the stress shielding effect. Some studies have confirmed that the microporous design of 3D-printed AVB can create an osteogenic environment suitable for bone morphogenetic protein 2 (BMP2), BMP4, and BMP7; promote the differentiation and maturation of osteoblasts; and facilitate earlier completion of osseous fusion (38, 39).

In addition, implant subsidence is also related to many other factors. Bone density is positively correlated with the maximum load on the vertebral endplate; therefore, patients with osteoporosis are more likely to experience subsidence (6). Bao et al. (40) found that preoperative or postoperative radiotherapy and resection of more than 2 vertebral body segments were independent risk factors for implants failure. Radiotherapy does not cause fracture of bone collagen but changes the biomechanical characteristics of the bone matrix, resulting in reduced fatigue resistance (41). Multi-segment resection can lead to insufficient blood supply to the surrounding tissues, which prolongs bone fusion time and affects spinal stability. Weber et al. (42) also noticed that excessive intervertebral space distraction increased the load between the implants and vertebral bodies, and the distraction height was positively correlated with the subsidence rate. Moreover, some studies have reported that the subsidence of implants may be related to the patient’s age, sex, and smoking status (6, 42). Since the included studies’ data are incomplete and have a low level of evidence, we are conducting a prospective and large-scale cases study to explore factors influencing implants subsidence after total en bloc spondylectomy and spinal reconstruction of spinal tumors.

When TES is performed for spinal tumors, normal tissue structures such as blood vessels and nerves are inevitably damaged during the operation, leading to severe trauma, prolonged operation, and multiple complications (43). Early complications mainly include pleural effusion, cerebrospinal fluid leakage, and infection, whereas late complications mainly include the failure of internal fixation systems, such as broken nails and broken rods. Based on our results, the incidence of early complications was significantly lower in the 3D-printed AVB group than in the conventional TMC group. We believe that this difference is not only related to the type of implants, but also to the experience of the surgeon and the preoperative patient’s physical condition. If not treated on time, early complications will affect the stability of implants fusion and spinal reconstruction and even cause late complications.

Although this study revealed some important discoveries, some limitations should be acknowledged. First, this was a systematic review and meta-analysis, and all nine studies included were non-randomized controlled trials with a low level of evidence. Second, only nine studies were included, all of which were from China; therefore, a certain publication bias existed. We performed sensitivity, subgroup, and publication bias analyses for the operation time and intraoperative blood loss, but sensitivity and subgroup analyses could not eliminate heterogeneity, and publication bias of intraoperative blood loss was found. Third, the results have a selection bias due to the differences in tumor type, location, size, number of vertebral bodies involved, and surgical approach. However, due to the insufficient number of included studies and missing data, we could only perform meta-regression analysis for the tumor type and location, but not the rest. Therefore, these limitations should be noted when generalizing the results. The results require further validation in the future as more high-quality studies become available.

## 5 Conclusion

As the first systematic review and meta-analysis comparing the efficacy and safety of 3D-printed vertebral bodies and conventional TMC in spinal reconstruction after total en bloc spondylectomy for spinal tumors, our findings demonstrate that 3D-printed AVB is superior to conventional TMC in reducing operation time, intraoperative blood loss, postoperative VAS score, and occurrence of vertebral body subsidence and early complications.

## Data availability statement

The original contributions presented in the study are included in the article/**Supplementary Material**. Further inquiries can be directed to the corresponding author.

## Author contributions

MD: Conceptualization, Investigation, Methodology, Writing – original draft, Writing – review & editing. YJG: Data curation, Writing – original draft. HF: Supervision, Writing – original draft. YW: Visualization, Writing – review & editing. JL: Formal analysis, Writing – original draft. JB: Supervision, Writing – original draft. PS: Methodology, Supervision, Writing – original draft. YG: Data curation, Methodology, Writing – original draft. ZL: Supervision, Validation, Writing – original draft. YF: Resources, Supervision, Validation, Visualization, Writing – original draft, Writing – review & editing.
