# Supplementary material for: Comparison of clinical efficacy of 3D-printed artificial vertebral body and conventional titanium mesh cage in spinal reconstruction after total en bloc spondylectomy for spinal tumors: a systematic review and meta-analysis
Source: Front Oncol. 2024 Feb 6;14:1327319. doi: 10.3389/fonc.2024.1327319 (PMC10878420; doi:10.3389/fonc.2024.1327319)

**Supplementary Figure 1.** Funnel plot of the operation time.


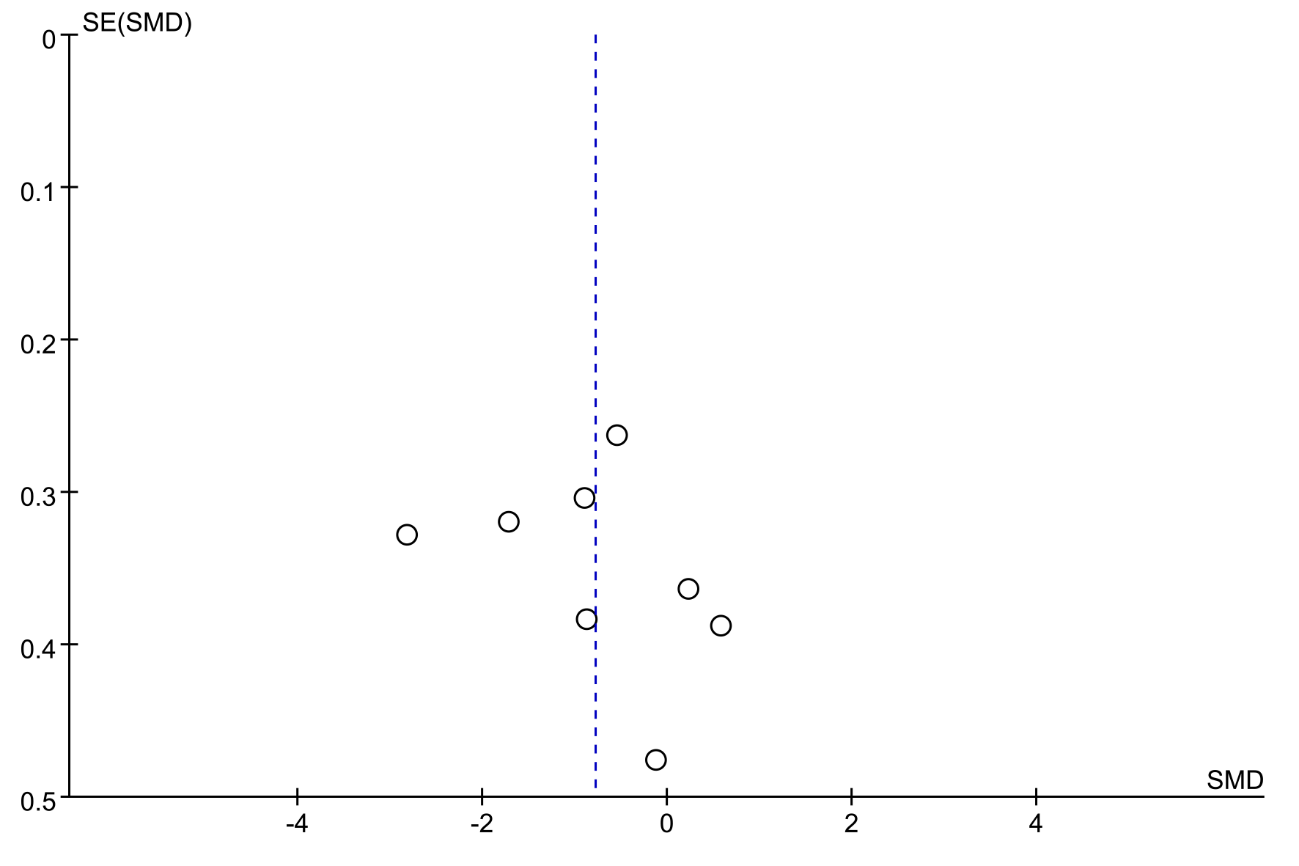


**Supplementary Figure 2.** Funnel plot of the intraoperative blood loss.


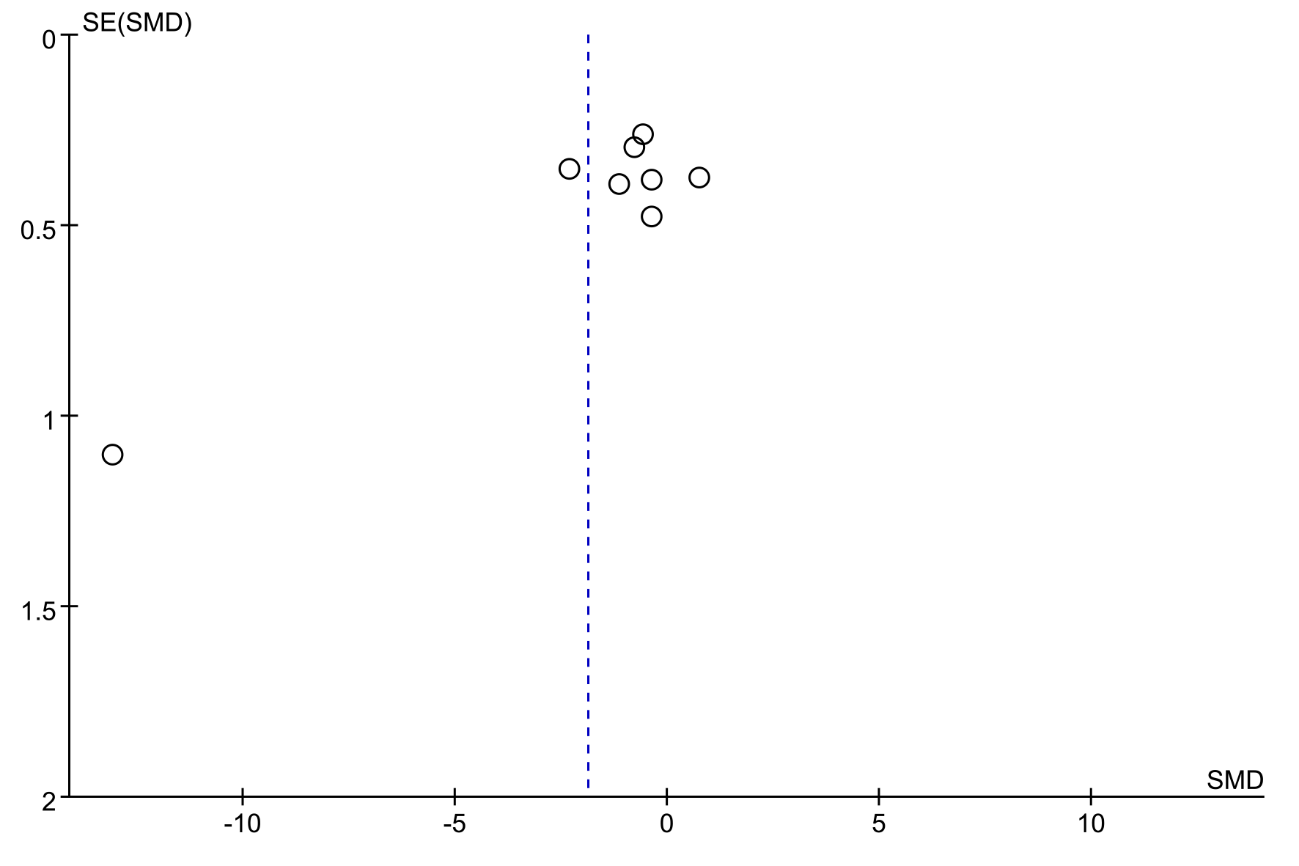


**Supplementary Figure 3.** Funnel plot of the preoperative and postoperative visual analogue scale scores.


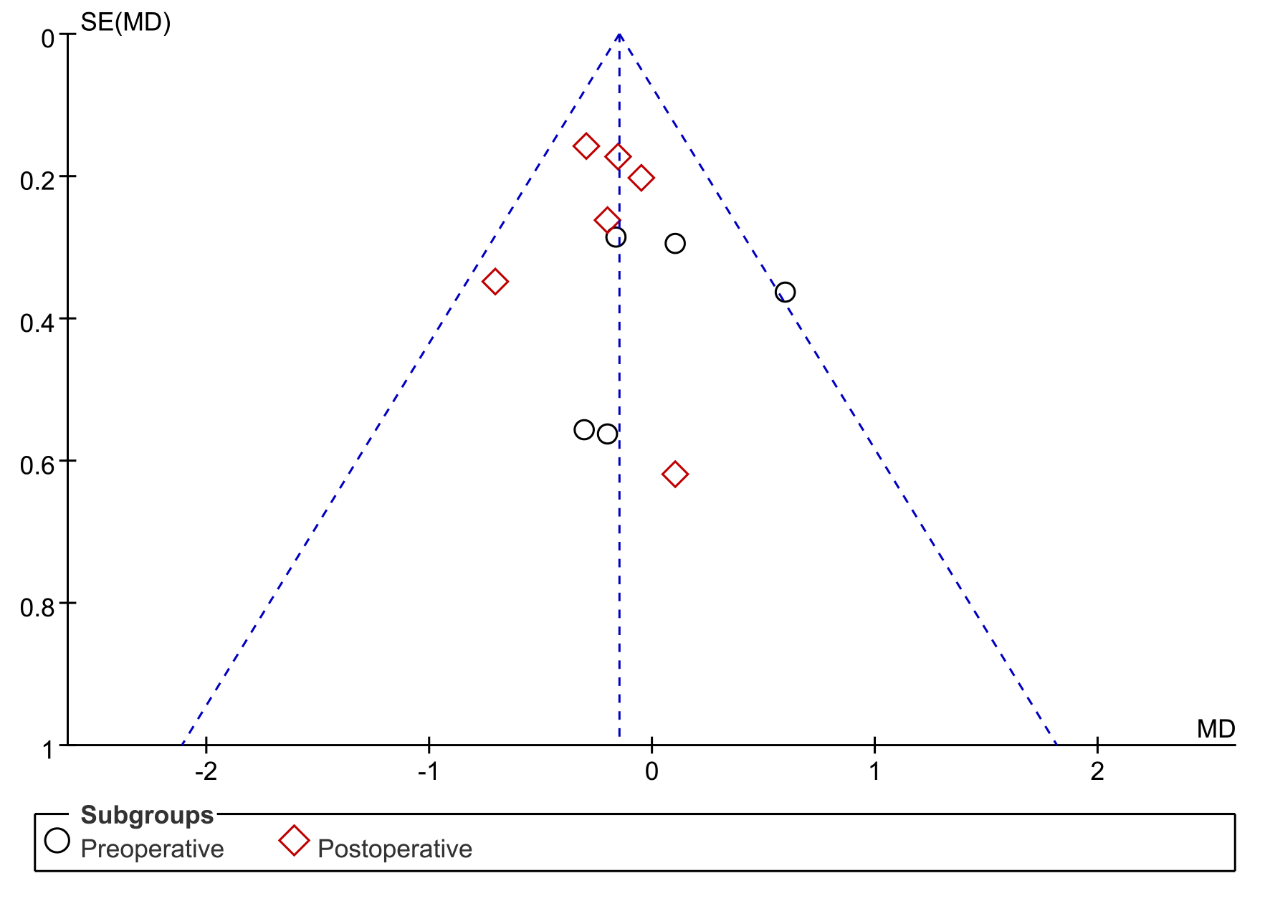


**Supplementary Figure 4.** Funnel plot of the preoperative and postoperative Frankel classification.


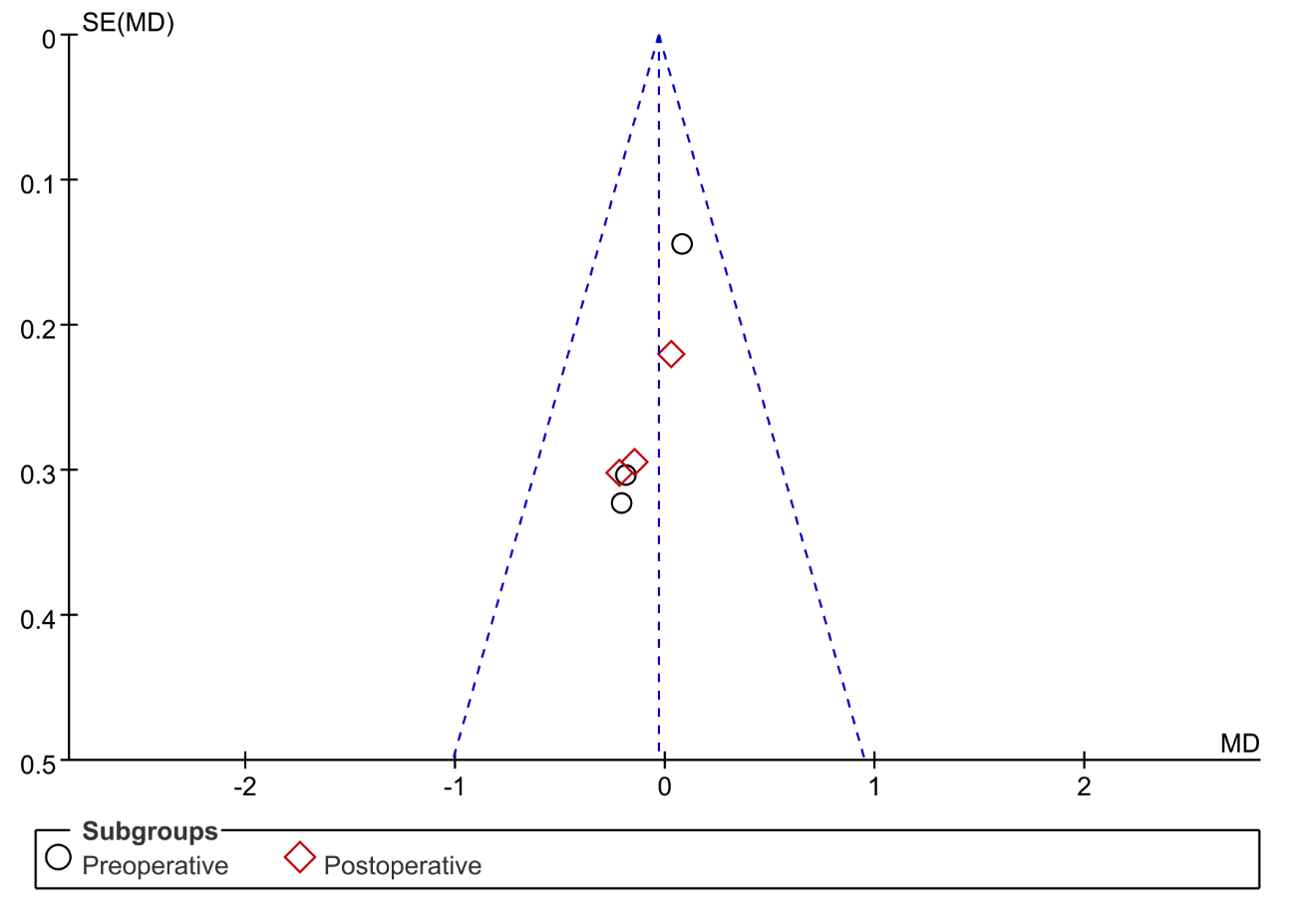


**Supplementary Figure 5.** Funnel plot of the occurrence of vertebral body subsidence.


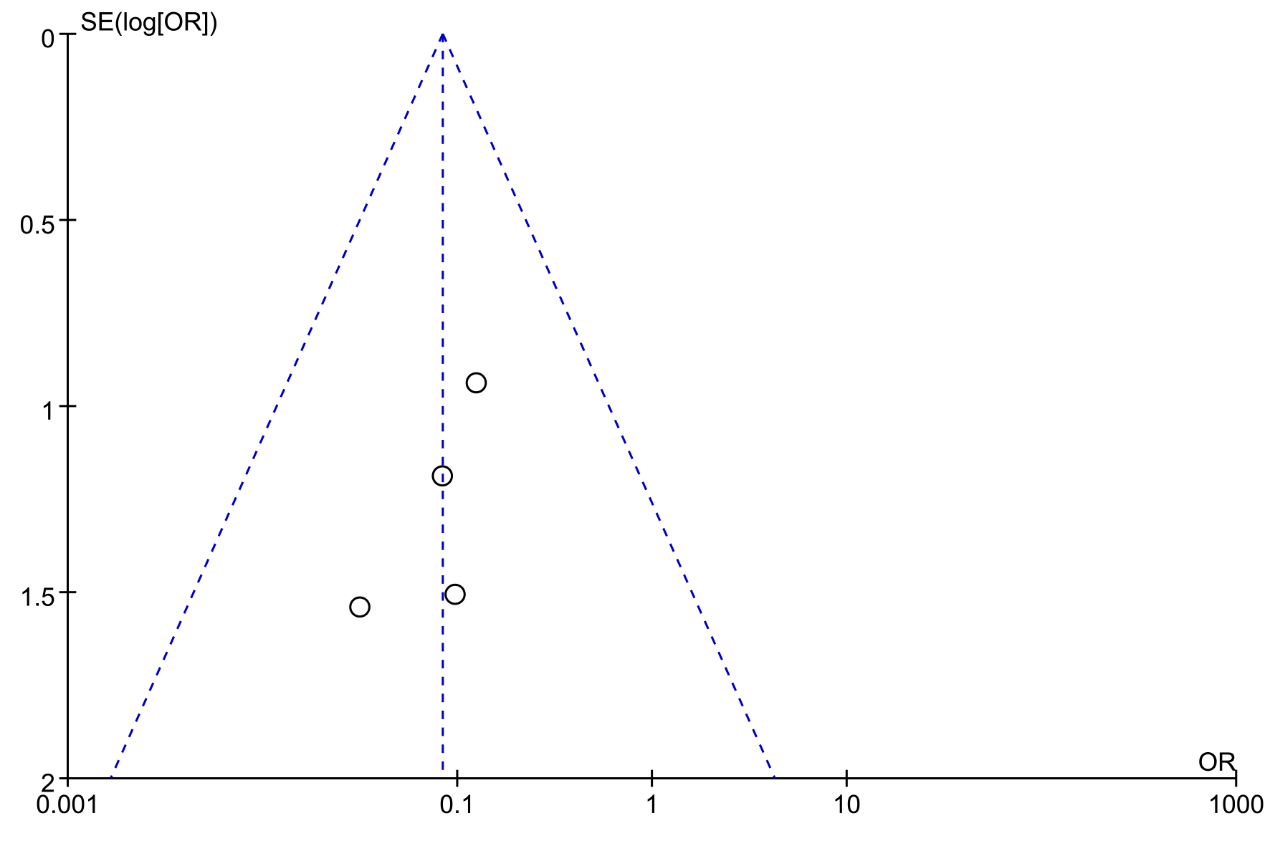


**Supplementary Figure 6.** Funnel plot of the occurrence of early complications.


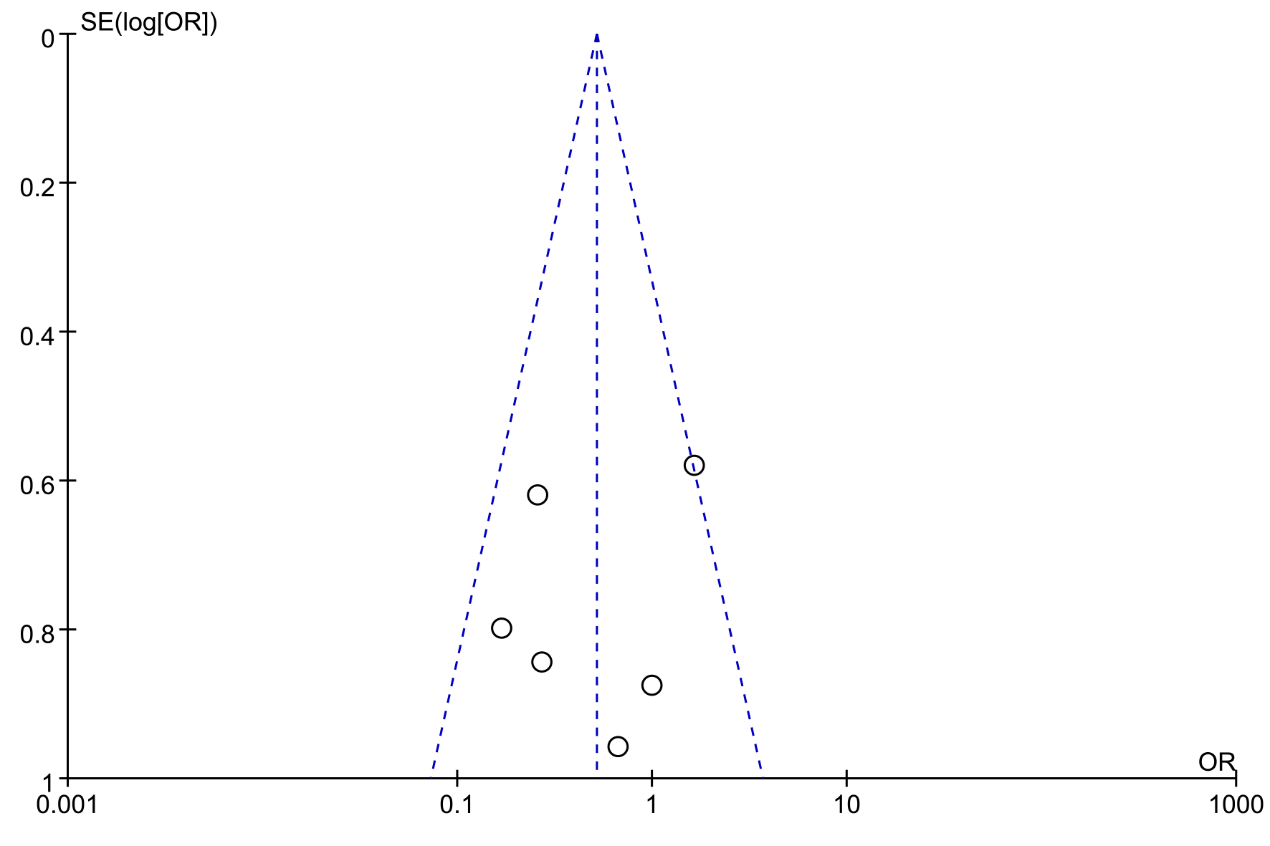


**Supplementary Figure 7.** Funnel plot of the subgroup analysis of the operative time.


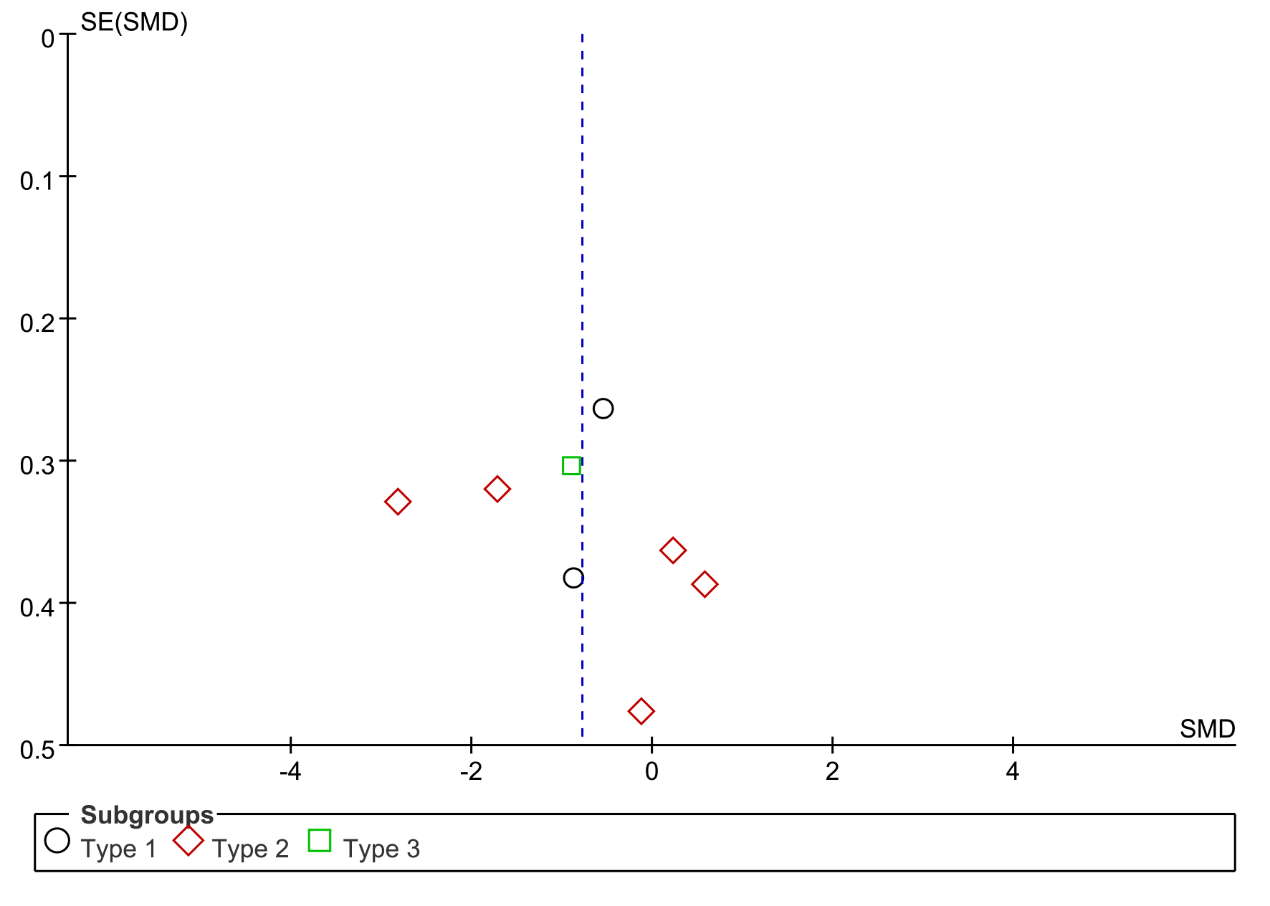


**Supplementary Figure 8.** Funnel plot of the subgroup analysis of the intraoperative blood loss.


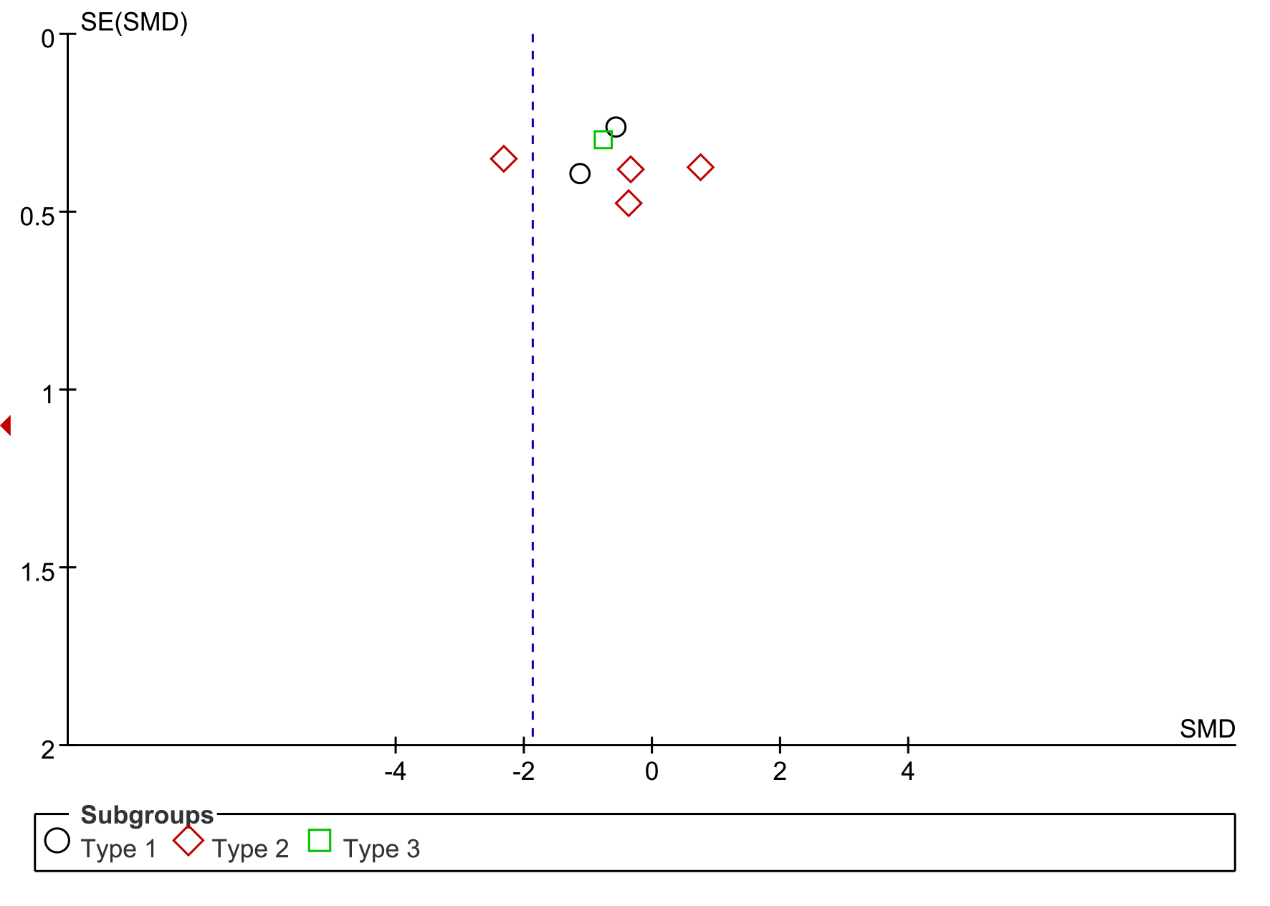

Supplement: Supplementary file 1 [file DataSheet_1.docx]
