# Supplementary material for: Comparison of clinical efficacy of 3D-printed artificial vertebral body and conventional titanium mesh cage in spinal reconstruction after total en bloc spondylectomy for spinal tumors: a systematic review and meta-analysis
Source: Front Oncol. 2024 Feb 6;14:1327319. doi: 10.3389/fonc.2024.1327319 (PMC10878420; doi:10.3389/fonc.2024.1327319)
Supplement: Supplementary file 2 [file Table_1.docx]

**Supplementary Table 1.** Tumor Pathology and Type in the Nine Included Studies.

| **Type** | **Pathological Diagnosis** | **Chen et al. (14)** | | **Hu et al. (15)** | | **Ji et al. (16)** | | **Li et al. (17)** | | **Qing et al. (18)** | | **Wang L et al. (19)** | | **Wang X et al. (20)** | | **Zhang et al. (21)** | | **Zhou et al. (22)** | |
| --- | --- | --- | --- | --- | --- | --- | --- | --- | --- | --- | --- | --- | --- | --- | --- | --- | --- | --- | --- |
|  |  | **AVB** | **TMC** | **AVB** | **TMC** | **AVB** | **TMC** | **AVB** | **TMC** | **AVB** | **TMC** | **AVB** | **TMC** | **AVB** | **TMC** | **AVB** | **TMC** | **AVB** | **TMC** |
| **Primary** | Aneurismal Bone Cyst | - | - | - | - | - | | - | - | 10 | 14 | - | - | NA | NA | - | 1 | - | - |
|  | Chondrosarcoma | - | - | - | - | - | | 1 | 4 |  |  | - | - |  |  | - | 1 | - | - |
|  | Chordoma | - | - | 8 | 6 | - | | 2 | - |  |  | - | - |  |  | - | - | 9 | 15 |
|  | Diffuse Large B-Cell Lymphoma | - | - | - | - | - | | - | - |  |  | - | - |  |  | 1 | - | - | - |
|  | Ewing Sarcoma | - | - | 1 | - | - | | - | - |  |  | - | - |  |  | - | - | - | - |
|  | Fibrous Dysplasia | - | - | - | - | - | | - | - |  |  | - | - |  |  | 1 | - | - | - |
|  | Giant Cell Tumor | 13 | 11 | 8 | 3 | 2 | | 2 | - |  |  | - | - |  |  | 2 | 2 | - | - |
|  | Hemangioendothelioma | - | - | - | - | - | | - | 1 |  |  | - | - |  |  | - | 2 | - | - |
|  | Hemangioma | 7 | 5 | - | - | 1 | | - | - |  |  | - | - |  |  | 3 | 2 | - | - |
|  | Hemangiopericytoma | - | - | - | 1 | - | | - | - |  |  | - | - |  |  | - | - | - | - |
|  | Malignant Histiocytosis | - | - | - | - | - | | - | - |  |  | - | - |  |  | - | 1 | - | - |
|  | Multiple Myeloma | - | - | - | - | 4 | | - | - |  |  | - | - |  |  | - | 1 | - | - |
|  | Osteoblastoma | - | - | - | 2 | - | | - | 1 |  |  | - | - |  |  | - | - | - | - |
|  | Osteosarcoma | 10 | 14 | - | - | 2 | | - | - |  |  | - | - |  |  | 4 | - | - | - |
|  | Paraganglioma | - | - | 1 | - | - | | - | - |  |  | - | - |  |  | - | - | - | - |
|  | Primitive Neurotodermal Tumour | - | - | - | - | 1 | | - | - |  |  | - | - |  |  | 2 | - | - | - |
|  | Schwannoma | - | - | - | 1 | - | | - | - |  |  | - | - |  |  | - | - | - | - |
|  | Solitary Fibroma | - | - | - | - | - | | - | 1 |  |  | - | - |  |  | - | - | - | - |
|  | Synovialsarcoma | - | - | - | - | - | | 2 | - |  |  | - | - |  |  | - | - | - | - |
| **Metastatic** | Breast Cancer | - | - | - | - | 3 | | - | - | 22 | 31 | 7 | 6 | NA | NA | - | 1 | - | - |
|  | Cervical Cancer | - | - | - | - | - | | - | - |  |  | - | - |  |  | 1 | - | - | - |
|  | Clear Cell Sarcoma | - | - | - | - | 1 | | - | - |  |  | - | - |  |  | - | - | - | - |
|  | Colon Cancer | - | - | - | - | - | | - | 1 |  |  | - | - |  |  | - | - | - | - |
|  | Gastric Cancer | - | - | - | - | 1 | | - | - |  |  | - | - |  |  | - | - | - | - |
|  | Kidney Cancer | - | - | - | - | 2 | | - | 1 |  |  | - | 1 |  |  | - | - | - | - |
|  | Liver Cancer | - | - | - | - | 4 | | - | - |  |  | 2 | 1 |  |  | - | - | - | - |
|  | Lung Cancer | - | - | - | - | 6 | | 1 | - |  |  | 9 | 10 |  |  | - | 2 | - | - |
|  | Neuroendocrine Tumor | - | - | - | - | - | | - | - |  |  | - | - |  |  | - | 1 | - | - |
|  | Pancreatic Cancer | - | - | - | - | 1 | | - | - |  |  | - | - |  |  | - | - | - | - |
|  | Parotid Gland Cancer | - | - | - | - | 1 | | - | - |  |  | - | - |  |  | - | - | - | - |
|  | Prostatic Cancer | - | - | - | - | 2 | | - | - |  |  | 4 | 3 |  |  | - | - | - | - |
|  | Rectum Cancer | - | - | - | - | 2 | | - | - |  |  | - | - |  |  | - | - | - | - |
|  | Thyroid Cancer | - | - | - | - | - | | - | 1 |  |  | 2 | 3 |  |  | - | - | - | - |
